# Mapping and decoding cortical engagement during motor imagery, mental arithmetic, and silent word generation using MEG

**DOI:** 10.1002/hbm.26284

**Published:** 2023-03-29

**Authors:** Vahab Youssofzadeh, Sujit Roy, Anirban Chowdhury, Aqil Izadysadr, Lauri Parkkonen, Manoj Raghavan, Girijesh Prasad

**Affiliations:** ^1^ Department of Neurology Medical College of Wisconsin Milwaukee Wisconsin USA; ^2^ BrainAlive Research Pvt Ltd Kanpur Uttar Pradesh India; ^3^ School of Computer Science and Electronic Engineering University of Essex Colchester UK; ^4^ Wake Forest School of Medicine, Winston‐Salem Winston‐Salem North Carolina USA; ^5^ Department of Neuroscience and Biomedical Engineering Aalto University School of Science Espoo Finland; ^6^ School of Computing, Engineering and Intelligent Systems Ulster University Londonderry UK

**Keywords:** beta‐decrements, brain–computer interface, classification, magnetoencephalography, task imagery

## Abstract

Accurate quantification of cortical engagement during mental imagery tasks remains a challenging brain‐imaging problem with immediate relevance to developing brain–computer interfaces. We analyzed magnetoencephalography (MEG) data from 18 individuals completing cued motor imagery, mental arithmetic, and silent word generation tasks. Participants imagined movements of both hands (HANDS) and both feet (FEET), subtracted two numbers (SUB), and silently generated words (WORD). The task‐related cortical engagement was inferred from beta band (17–25 Hz) power decrements estimated using a frequency‐resolved beamforming method. In the hands and feet motor imagery tasks, beta power consistently decreased in premotor and motor areas. In the word and subtraction tasks, beta‐power decrements showed engagements in language and arithmetic processing within the temporal, parietal, and inferior frontal regions. A support vector machine classification of beta power decrements yielded high accuracy rates of 74 and 68% for classifying motor‐imagery (HANDS vs. FEET) and cognitive (WORD vs. SUB) tasks, respectively. From the motor‐versus‐nonmotor contrasts, excellent accuracy rates of 85 and 80% were observed for hands‐versus‐word and hands‐versus‐sub, respectively. A multivariate Gaussian‐process classifier provided an accuracy rate of 60% for the four‐way (HANDS‐FEET‐WORD‐SUB) classification problem. Individual task performance was revealed by within‐subject correlations of beta‐decrements. Beta‐power decrements are helpful metrics for mapping and decoding cortical engagement during mental processes in the absence of sensory stimuli or overt behavioral outputs. Markers derived based on beta decrements may be suitable for rehabilitation purposes, to characterize motor or cognitive impairments, or to treat patients recovering from a cerebral stroke.

## Graphical abstract

We analyzed magnetoencephalography data from 18 individuals completing cued motor imagery, mental arithmetic, and silent word generation tasks. Task‐related cortical engagement was inferred from beta‐band power decrements estimated using a frequency‐resolved beamforming method. In the hands and feet motor imagery tasks, beta power consistently decreased in premotor and motor areas. In the word and subtraction tasks, beta‐power decrements showed engagement in language and arithmetic processing within the temporal, parietal, and inferior frontal regions. Beta‐power decrements are useful metrics for mapping and decoding cortical engagement during mental processes in the absence of sensory stimuli or overt behavioral outputs.

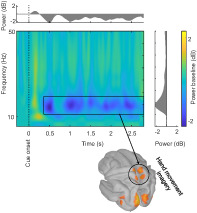

## 1 INTRODUCTION

Tasks designed for the development of brain–computer interfaces (BCI) often involve mental simulation of actions without their actual execution (Crammond, 1997; Jeannerod, 1994; Szameitat et al., 2007). In contrast to mapping brain responses driven by external sensory stimuli, the mapping of brain areas activated by such tasks remains a key challenge in BCI research (Wolpaw et al., 2000), both due to the distributed nature of brain networks that are engaged during cognitive tasks and the limitations of different imaging methods. The high spatial and temporal resolution of magnetoencephalography (MEG) makes it inherently suitable for mapping the dynamical engagement of brain areas during mental processes, thus the recent interest in MEG in the context of BCI research (Lai et al., 2005; Mellinger et al., 2007; Parkkonen, 2015).

Specific aspects of task‐induced changes in rhythmic cerebral electrical activity are increasingly recognized as signatures of local cortical engagement during information processing in the brain (Engel & Fries, 2010; Giraud & Poeppel, 2012; Hauk et al., 2017; Lewis & Bastiaansen, 2015; McFarland et al., 2000; Meyer, 2018; Pfurtscheller & Neuper, 1997; Salmelin & Hari, 1994; Schnitzler et al., 1997; Spitzer & Haegens, 2017). Induced oscillatory responses are loosely time‐locked but not strictly phase‐locked to stimuli, and therefore cannot be extracted by averaging the time‐domain responses, but they may be detected as power changes in different frequency bands (Başar & Bullock, 1992; David et al., 2006; Pfurtscheller & Andrew, 1999; Tallon‐Baudry, 1999). Over the last few decades, a number of studies using EEG, electrocorticography (ECoG), and MEG have demonstrated increases in gamma band (>40 Hz) power concurrent with a decrease in power in the alpha (8–12 Hz) and beta (13–30 Hz) bands as a characteristic feature of the cortical response to afferent stimuli (Crone et al., 1998, 2006; Eulitz et al., 1996; Miller et al., 2007; Pfurtscheller, 1991; Singh et al., 2002; Wagner et al., 2012). The gamma‐band activity appears to be related to local neuronal populations' firing rates (Edwards et al., 2005; Manning et al., 2009; Michalareas et al., 2016; Nir et al., 2007; Ray & Maunsell, 2011) and is more focally expressed than concomitant decreases in alpha‐ and beta‐band power (Crone et al., 1998, 2006; Eulitz et al., 1996; Miller et al., 2007; Pfurtscheller, 1991; Singh et al., 2002; Wagner et al., 2012). The high spatiotemporal resolution of MEG is ideally suited to capturing such time–frequency dynamics and localizing their cortical sources (Gross, 2019).

While gamma‐band activity arising outside primary sensory or motor cortices may be less readily detectable in M/EEG (e.g., compared to ECoG), task‐related power decrements in the beta‐band consistent with the expected cortical engagement have been demonstrated by several studies (Neuper & Pfurtscheller, 2001; Seeber et al., 2014; Weiss & Mueller, 2012; Youssofzadeh et al., 2020). Importantly, decreased beta‐band power has been observed in motor imagery (Halme, 2019; Klepp et al., 2015; Kraeutner et al., 2014; Pfurtscheller et al., 2006) as well as during various cognitive tasks (Armeni et al., 2019; Lewis & Bastiaansen, 2015; Weiss & Mueller, 2012). For example, beta‐band power decreases have been found to be correlated with imaginary foot movements when walking on a virtual street (Neuper & Pfurtscheller, 2001; Pfurtscheller et al., 2006). A pioneering EEG‐BCI study showed that feature values based on beta‐band activity over the sensorimotor area provided the largest discrimination, >90% and 80% for execution and imagination, respectively (Bai et al., 2008). On the other hand, the suppression of beta during language processing has been associated with novel or unexpected stimuli (Bastiaansen et al., 2010; Engel & Fries, 2010; Weiss & Mueller, 2012), semantically incongruous sentences (Luo et al., 2010; Wang et al., 2012), and unexpected high‐versus‐low perplexity (Armeni et al., 2019). Many M/EEG studies support the utility of monitoring beta‐band power in the localization of language functions in healthy controls and patients (Findlay et al., 2012; Fisher et al., 2008; Grabner et al., 2007; Kadis et al., 2008; Passaro et al., 2011; Weiss & Mueller, 2012; Youssofzadeh et al., 2020).

In this study, we estimated task‐induced beta‐band (17–25 Hz) power decrements from MEG recordings performed during movement‐imagery of the hands or feet, mental arithmetic, and silent word generation in a group of healthy individuals (*N* = 18). Silent word generation and mental arithmetic tasks are both examples of cognitive tasks that do not involve motor activity but require the activation of language and numerical processing regions in the brain, respectively. By comparing brain activity during these tasks with brain activity during motor imagery tasks, we investigated the unique neural signatures of the task imagery conditions. Such comparisons can help to clarify the specific brain regions and processes involved in motor imagery and to distinguish it from other types of mental activity. For instance, if the same brain regions are active during both a motor imagery task and a silent word generation task, this suggests that these regions may be involved in more general processes, such as attention or working memory. In our work, we demonstrate that source‐space task‐related beta‐band power decrements can map cortical engagement during task imagery processes. This is supported by substantially high classification accuracy and consistent regions of interest (ROIs) suggested by the classification weight maps. For ease of reporting, we use the term “beta decrements” to refer to decreased source power in the beta‐band frequency relative to the pre‐cue baseline period (also known as, event‐related desynchronization, ERD, or suppression) throughout the paper.

## 2 MATERIALS AND METHODS

### 2.1 Participants

We analyzed MEG data from 18 participants who performed cued motor imagery, mental arithmetic, or word generation during MEG recording. Demographic details of the participants have been reported previously (Rathee et al., 2021). They were recruited from the community through advertising or were students and staff of the University of Ulster; all had normal hearing and normal or corrected‐to‐normal vision. Individuals with a history of neurological or psychiatric illness and individuals taking psychoactive medication were excluded. The participants were 15 men and 3 women, with a mean age of 28.56 years ± 5.7 (SD), 16 right‐handed, and 2 left‐handed, based on the self‐reported questionnaire. The participants signed a written consent form before commencing the experiments. The University of Ulster, Northern Ireland, UK's ethics committee approved the experimental protocol.

### 2.2 Data acquisition

MEG data were recorded using a 306‐channel (204 planar gradiometers and 102 magnetometers) whole‐head neuromagnetometer system (Elekta Neuromag TRIUX; MEGIN Oy, Helsinki, Finland) in the upright position in a magnetically shielded room (ETS‐Lindgren, Eura, Finland) located at the University of Ulster, Magee campus, Northern Ireland, UK. The raw data were acquired at a sampling rate of 1 kHz and high‐pass filtered with a cutoff frequency of 0.03 Hz. The position of the participant's head relative to the sensors was determined using four head‐position indicator coils attached to the scalp surface. Three anatomical landmarks (nasion and left and right pre‐auricular points) and the head shape were digitized using a Polhemus Fastrak system (Polhemus; Colchester, VT) for alignment with the template MRI. The cues for the tasks were displayed on a projector screen (a Panasonic projector with a screen resolution of 1024 × 768 pixels and a refresh rate of 60 Hz). Participants were seated in a comfortable chair approximately 80 cm from the projector screen. Further details can be found in an earlier paper (Rathee et al., 2021; Roy et al., 2020).

### 2.3 MEG task paradigm

**FIGURE 1:**
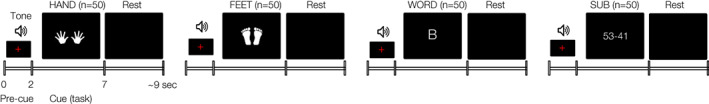
Brain–computer interfaces (BCI) task paradigm. Participants took part in two sessions of a task‐imagery (BCI) magnetoencephalography (MEG) experiment. The task consisted of two motor‐ (Hands and Feet movements) and two cognitive (Word generation and mathematical Subtraction) tasks. A red fixation cross (“+”) and a tone were presented during the pre‐cue period. During a cue‐task period, a picture was presented corresponding to each of the four imagery task conditions of HANDS, FEET, WORD, and SUB. During the HANDS condition, participants imagined grasping with hand movement. During the FOOT, participants imagined the movement of the foot upward. During the WORD, participants generated a word starting with a letter shown on the screen. The letters were randomly selected from A to Z. During the SUB condition, participants completed a two‐digit against two‐digit subtraction task.

Participants completed two sessions of a BCI experiment during MEG recording. Sessions were recorded on different days for each participant. The paradigm required the completion of four mental tasks when the appropriate cue was presented visually: imagining the movements of both hands (HANDS), the movements of both feet (FEET), mental subtraction of two digits (SUB), and the generation of a word starting with the cued letter (WORD), as illustrated in Figure 1. Each task trial started with a pre‐cue period lasting ~2 s, then the appearance of a static visual cue, which remained on the screen for 5 s while the participant performed the cued task, followed by 1.5–2 s of rest. No feedback was provided while participants completed the experiment.

During the pre‐cue period, a red fixation cross (“+”) was presented along with an auditory tone (500 Hz). During the cued‐task period, a visual cue was presented corresponding to each of the four tasks (HANDS, FEET, WORD, or SUB). During the HANDS condition, the cue was a picture of two hands, and participants imagined grasping with both hands. During the FEET condition, the cue was a picture of two feet, and participants imagined dorsiflexing both feet. During the SUB condition, a subtraction problem (the subtraction of a two‐digit from a two‐ or three‐digit number) was displayed on the screen for the participant to mentally execute. During the WORD condition, participants mentally generated a word starting with the letter cue shown on the screen, for example, BOY for the letter B. The letters were randomly selected from the alphabet, A‐Z (*N* = 26). For each task condition, 50 trials were acquired for a total of 400 trials (2 × 50 × 4, corresponding to the session, trial, and task conditions) per subject. The order of the tasks was randomized within each session.

To control for the implicit nature of motor imagery tasks, we employed several strategies to ensure that participants were engaging in the task as instructed. Strategies include Verbal confirmation: Participants were asked to verbally confirm that they were imagining the movement as instructed and not performing the movement. Visual supervision: Participants were monitored visually during the task to ensure that they were not physically moving their limbs or other parts of their bodies. Instruction repetition: Participants receive repeated and clear instructions on how to perform the task and what is expected of them, to reduce any confusion and increase task compliance. Practice trials: Participants were given practice trials to familiarize themselves with the task and reduce any performance anxiety. With these, we hoped to increase the likelihood that participants were engaging in the motor imagery task as instructed and not physically performing the movement.

Following each recording session, participants were asked about their level of engagement in the task, such as their confidence in their ability to perform the task and the degree to which they felt they were imagining the movement. They were also asked for detailed information about any distractions or the difficulty of the task.

### 2.4 Data analysis

We analyzed the MEG responses following cue onset (i.e., the period corresponding to task performance) using six nonoverlapping 400‐ms temporal windows (from 400 to 2800 ms). The initial 400 ms after cue onset was excluded in order to discard transient sensory responses to the cue onset. We compared responses during these temporal windows to a 400‐ms period immediately preceding the cue onset, which served as a baseline (see Section 2.5 for details).

#### 2.4.1 Data preprocessing

Head localization was measured before and after the MEG sessions to assess the head movements that may have occurred during the measurements. Using MaxFilter software ver. 2.2 (MEGIN Oy, Helsinki, Finland), a temporal variant of signal space separation was applied to suppress external magnetic interference (software shielding), compensate for signal distortions caused by head movements, and normalize head positions (Taulu & Simola, 2006).

Data were epoched from −1 to 4 s relative to the cue onset, then band‐pass‐filtered (Butterworth with an order of 4) to 1–40 Hz. Trials containing artifacts (signal jumps, eye blinks, or muscle contractions) were removed by a threshold value defined by a variance exceeding 3 × 10^−24^ *T*, a kurtosis larger than 15, and a z‐score larger than 4. Cardiac artifacts were inspected and removed via independent component analysis using the infomax algorithm (Bell & Sejnowski, 1995). An average (±SD) of 8 ± 6 trials per session was omitted. Note that we aimed at a modest (conservative) rejection of trials since our analysis was focused on the beta‐band frequency, which is less affected by the low‐ and high‐frequency artifacts.

**FIGURE 2:**
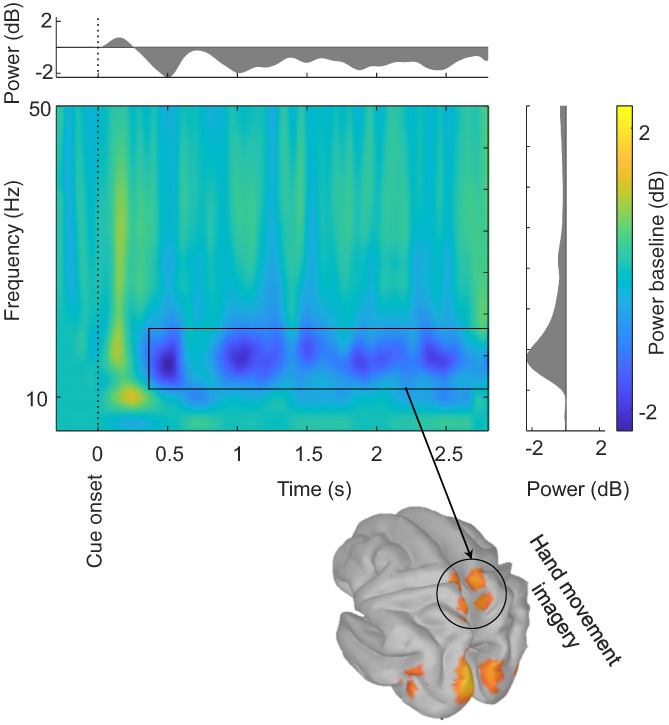
Time–frequency representation of a hand movement imagery task. An average of all sensor‐level event‐related power changes relative to baseline (−300, 0 ms) was utilized to support the selection of beta‐band frequency, as specified by a rectangle. For illustrative purposes, the corresponding beta‐decrement source activities of an individual completing a hand movement motor imagery task are shown. The mean power sensor values of the global peak in time (500 ms) and frequency (18 Hz) are shown in the top and right plots, respectively, for inspection. For the source analysis, data trials in six nonoverlapping 400‐ms temporal windows (from 400 to 2800 ms) in a frequency range of 17–25 Hz were analyzed against a 300‐ms (−300 to 0 ms) prestimulus baseline.

A time–frequency representation (TFR) analysis of sensor‐level data was conducted to inspect the presence of beta‐power changes. The TFR analysis was conducted using multitapers in the range of 1–50 Hz. Using sensors, a frequency‐dependent sliding time window was analyzed in a time and frequency range of −400 ms to 3 s, and a three‐cycle‐long Hanning window (Δ*T* = 3/f, f is the frequency of interest) was used. Fourier representation was estimated using a spectral smoothing of Δ*F* = 0.8x *f*. The TFRs were baseline‐corrected based on the 400‐ms pre‐cue data. A sample TFR from an individual completing the HANDS imagery task is shown in Figure 2.

### 2.5 Frequency‐domain beamforming source analysis

The MEG data were coregistered to a T1‐weighed anatomical MRI template (ICBM152). Cortical surface reconstruction was performed on cortical pial surfaces, downsampled to 15,002 vertices. Overlapping spheres were used as a head model to estimate the lead‐field matrix. Beta‐band source power was estimated using Dynamic Imaging of Coherent Sources (DICS), a frequency‐resolved spatial filtering beamforming technique (Gross et al., 2001). DICS uses the data covariance matrix to calculate the spatial filter based on the sensor‐level cross‐spectral densities (CSD), and the filter is then applied to the sensor‐level CSD to reconstruct the source‐level CSDs of the pairwise voxel activations. This provides coherency measures between the source pairs (off‐diagonal elements of CSD) and the source power spectrum measures (diagonal elements of CSD). Our analysis only utilized the source power spectrum estimates of cortical activations of the BCI tasks.

Sensor‐level CSD data were estimated in a beta‐band frequency range of 17–25 Hz, using a center frequency of 21 Hz and a spectral smoothing window of 4 Hz. The Fourier transform and multitapering, with multiple tapers from Discrete Prolate Spheroidal Sequences, were used to estimate the CSDs (Slepian & Pollak, 1961). Beta‐band source power during the task‐performance period was contrasted against the 400‐ms pre‐cue baseline. To avoid biases due to unequal data segments, post‐cue data were analyzed using 6 nonoverlapping 400‐ms time windows, ranging from 400 ms (to control for sensory responses) to 2800 ms. The average of source intervals was used as the representative beta‐band power effects in each session. This resulted in a source map per task, per session, and per participant. For mapping purposes, the average of the two sessions was used to represent the task conditions for each individual. Individual source findings were evaluated session‐wise using machine learning and correlation analyses.

### 2.6 Group source analysis

A nonparametric permutation test was conducted to achieve a group‐level source analysis (Nichols & Holmes, 2002). An independent sample *t*‐test was conducted against a null hypothesis to derive the *t*‐statistics of each task condition. A Monte Carlo permutation test was applied with 5000 randomizations of extreme statistics. The extreme (maximum) statistics control the expected proportion of false positives (also known as, multiple comparisons). A critical alpha of .05 was applied to the permutation distribution to report the significant statistical effects.

**FIGURE 3:**
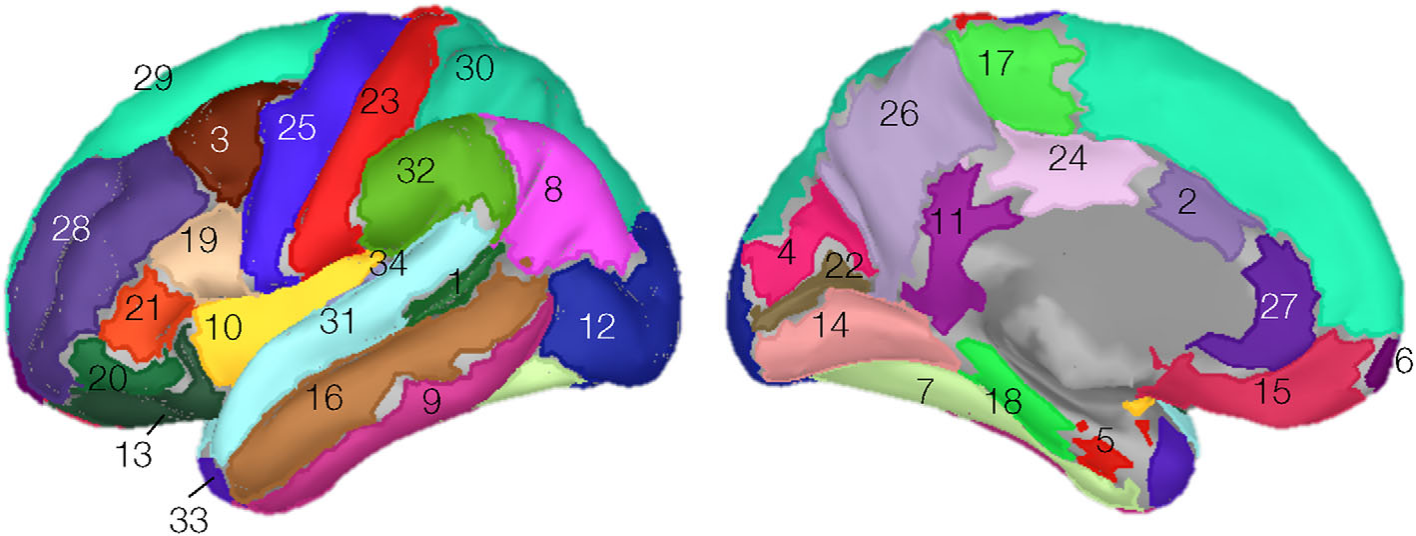
Desikan–Killiany surface atlas. A Desikan–Killiany atlas consisting of 68 (34 × 2) cortical regions distributed with Freesurfer (surfer.nmr.mgh.harvard.edu) was used to summarize source and pattern classification analyses. The regions were 1. bankssts; 2. caudal anterior cingulate, 3. caudal middle frontal; 4. cuneus; 5. entorhinal; 6. frontal pole; 7. fusiform; 8. inferior parietal; 9. inferior temporal; 10. insula; 11. isthmus cingulate; 12. lateral occipital; 13. lateral orbitofrontal; 14. lingual; 15. medial orbitofrontal; 16. middle temporal; 17. paracentral; 18. parahippocampal; 19. pars opercularis; 20. pars orbitalis; 21. pars triangularis; 22. pericalcarine; 23. postcentral; 24. posterior cingulate; 25. precentral; 26. precuneus; 27. rostral anterior cingulate; 28. rostral middle frontal; 29. superior frontal; 30. superior parietal; 31. superior temporal; 32. supramarginal; 33. temporal pole; 34. transverse temporal. Regions are randomly color‐coded.

The Desikan–Killiany (DK) atlas, consisting of 68 cortical regions (34 specific areas in L&R hemispheres) was used to summarize the power source measures (t‐values) across regions (Desikan et al., 2006). The color‐coded atlas regions are shown in Figure 3. ROIs with corrected *t*‐values with *p* < .05 were reported as being crucial to the task. Following the approach suggested by prior MEG studies (Papanicolaou et al., 2004; Raghavan et al., 2017; Tanaka et al., 2013; Youssofzadeh & Babajani‐feremi, 2019), a conventional laterality index, LI = (*L* − *R*)/(*L* + *R*), was computed for the *t*‐value of the left (*L*) and the right hemispheric (*R*) parcels to characterize the hemispheric involvement. LIs greater than 0.1 were considered left‐lateralized, those less than −0.1 were considered right‐lateralized, and intermediate ones were considered symmetric or bilateral.

### 2.7 Pattern classification analysis

A pattern classification analysis was conducted to assess the discriminability of beta decrements associated with the different tasks. Linear kernels per task condition were extracted as feature values from beta‐decrements across the cortex. Linear kernels are pairwise similarity measures (dot product) between task source activations that are summarized in a kernel matrix (*N* × *N* dimensions, *N*: 18 × 2 subjects and sessions) (LaConte et al., 2005; Schrouff et al., 2013). To achieve an unbiased classification, kernels were mean‐centered and normalized ($f_{i}=\left(f_{i}-\bar{f_{i}}\right)/\sigma_{i}$, $\bar{f_{i}}$, and $\sigma_{i}$ are the mean and standard deviation of *i*th feature, respectively). A linear support vector machine (SVM) with a default penalty parameter of *C* = 1.0 was applied to solve binary classification problems of HANDS‐versus‐FEET and WORD‐versus‐SUBtraction (Cortes & Vapnik, 1995). The SVM classifier relies on the assumption that two classes are separable by a linear decision boundary (separating hyperplane) in a feature space. In addition, a Gaussian process classifier (GPC) was used to classify all four BCI task conditions. The GPC is a probabilistic classification method relying on random field theory (Rasmussen & Williams, 2006) and has been successfully tested for the decoding of fMRI data (Marquand et al., 2010). For classification analysis, class and balanced accuracy (BA, an average of sensitivity and specificity) were reported (Schrouff et al., 2013). The accuracy of the SVM and GPC models was evaluated by a leave‐one‐participant‐out cross‐validation procedure, and *p*‐values were generated based on permutation testing with 1000 iterations. The DK surface atlas was used to summarize ROIs from generated SVM and GPC weight maps. The weight maps are the spatial representation of the decision function and define the level of voxel contributions to the classification process. To summarize ROI contributions, the voxel weights were averaged within the defined anatomical regions by taking the sum of their absolute values and dividing them by the size of the region. We report classification results and weight maps using combined two‐session beta decrements. Our initial examinations based on permutation analysis suggested no significant classification rates for single‐session data, likely due to the low sample size.

### 2.8 Correlation analysis

We performed two types of correlation analyses to assess the reproducibility of task‐related cortical engagement in individual subjects. We first asked how consistently, from session to session, a subject's task‐related activation conforms to the average map for the group for any particular task. To address this, we examined the correlation between beta‐decrement maps for each task and the class‐mean beta‐decrement maps for the two sessions. The correlation was measured using a nonparametric linear bivariate Spearman test. The class mean was defined as the average beta‐decrement map for all subjects across both sessions. These class‐mean beta‐decrements served as spatial templates for each task condition, against which the individual subject's map was compared. Second, we ask, how reproducible is a given subject's activation map from session to session for any given task? To address this, we computed the direct between‐session correlations of beta‐decrements across the brain for each subject and task condition. We examined both absolute correlations (session 2 vs. 1) and their ratios (maximum/minimum). We hypothesized that a higher correlation to the class means and a higher correlation of activations across sessions indicate a higher level of engagement with the tasks. We also hypothesized that the consistency of the correlation between a subject's activation map and the group mean across sessions may also identify the tasks for which activation maps are more stable.

## 3 RESULTS

All participants confirmed their high level of engagement in the task, and none reported the presence of any distractions or the difficulty of the task.

### 3.1 Group‐level source analysis of beta‐decrements

**FIGURE 4:**
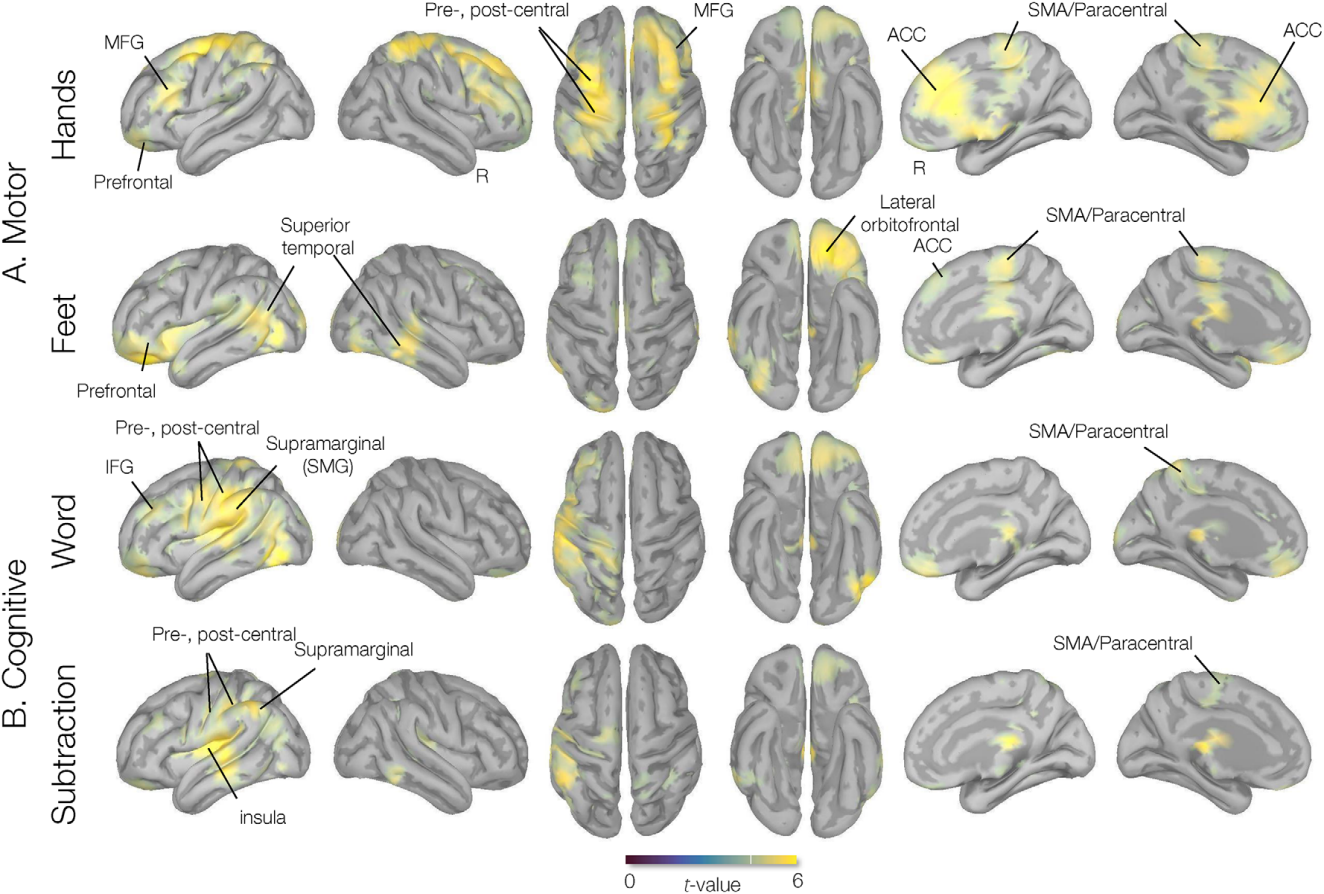
Group source analysis of task‐imagery brain–computer interfaces (BCI) experiment. Activation maps corresponding to (a) motor, hands and feet motor imagery, and (b) cognitive, word‐generation, and subtraction imagery tasks measured by the Dynamic Imaging of Coherent Sources (DICS) beamformer source analysis in a frequency range of beta (17–25 Hz) from 18 participants. *t*‐Maps are thresholded at a whole‐cortex corrected *p* < .05.

Source‐level MEG activity during HANDS and the FEET motor imagery tasks showed significant beta‐decrements in several cortical areas, including the precentral (the supplementary motor area [SMA]), the postcentral, and the anterior cingulate gyri (Figure 4a). Based on the laterality index, both tasks showed symmetric activation across the cerebral hemispheres, with LI_HANDS_ = 0.02 and LI_FEET_ = 0.04 (∣LI∣<0.1). Source‐level MEG activity during both the WORD and SUB task conditions showed significant beta‐decrements in the temporal (superior temporal), parietal (supramarginal), and (inferior) frontal regions (Figure 4b). The laterality indices indicate a left‐hemispheric dominance for both these tasks, with LI_WORD_ = 0.30 and LI_SUB_ = 0.26 (LI > 0.1).

**FIGURE 5:**
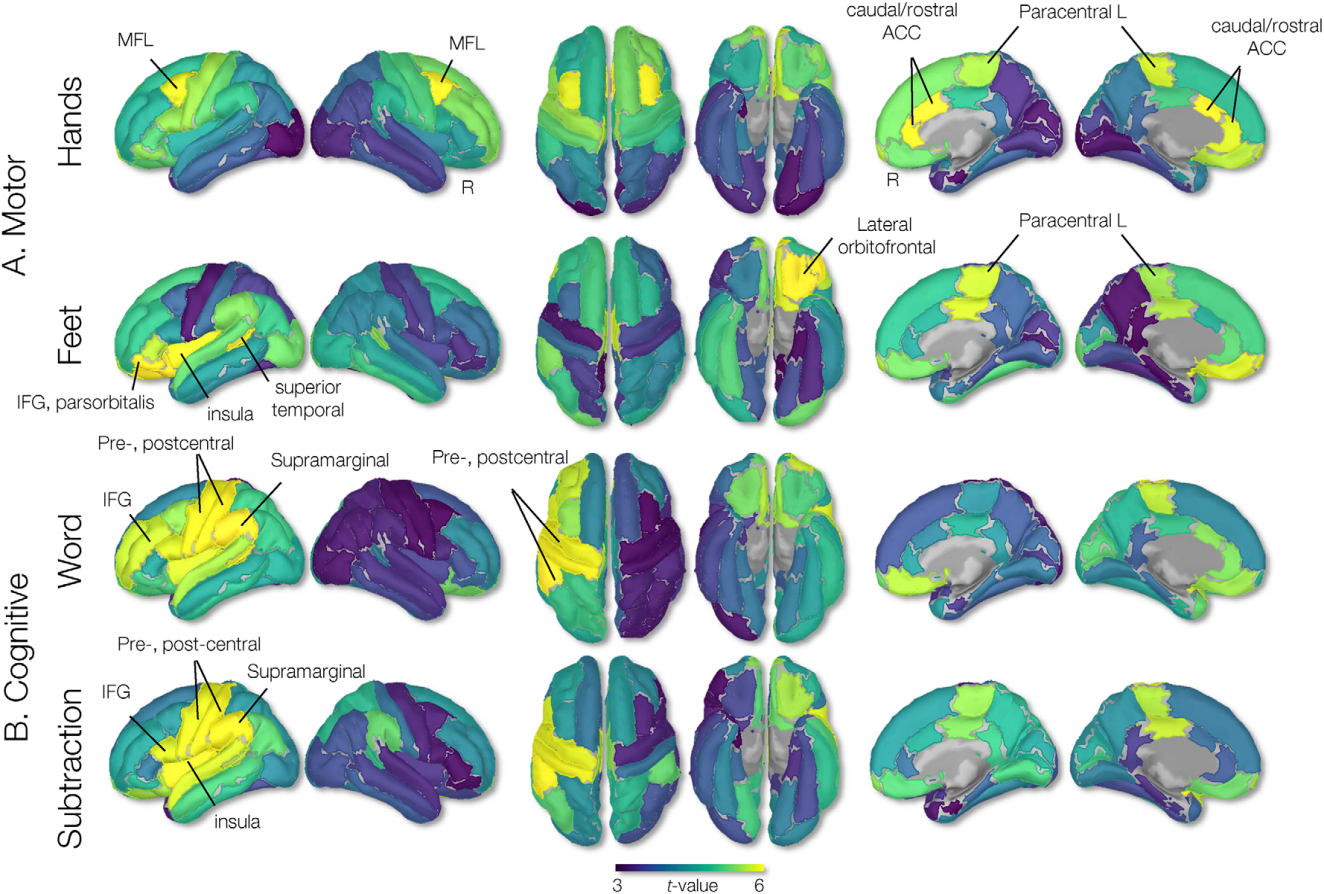
Group source parcellation analysis of task‐imagery brain–computer interfaces (BCI) experiment. Parcellation was conducted using a Desikan–Killiany atlas (Figure 3). Yellow and blue color‐coded areas represent higher and lower beta‐decrements, respectively, consistent with *t*‐values in Figure 4.

The parcellation analysis of the HANDS task showed prominent cortical engagements bilaterally in the caudal anterior cingulate, middle frontal gyri, and paracentral regions. The FEET task showed cortical activations bilaterally in the paracentral (SMA), lateral orbitofrontal, banks of the superior temporal sulcus, and left precentral regions (Figure 5a). The WORD task showed prominent left‐hemispheric cortical engagements in the supramarginal, postcentral, precentral, and inferior frontal gyri (IFG) and the pars opercularis, while the SUB task revealed left‐hemispheric cortical engagements within the temporal (transverse temporal and insula), parietal (supramarginal), and prefrontal (IFG, pars opercularis) regions (Figure 5b, second row). The ROIs are summarized in Table 1.

**TABLE 1:** ROI involved in four task imagery BCI activities, as measured by beta‐decrements

HANDS, ROI	*t*‐Value	FEET, ROI	*t*‐Value	WORD, ROI	*t*‐Value	SUB, ROI	*t*‐Value
Caudal anterior cingulate R	5.25	Lateral orbitofrontal l	5.06	Supramarginal L	5.04	Transverse temporal L	5.54
Caudal anterior cingulate L	4.81	Pars orbitalis l	4.89	Transverse temporal L	4.96	Insula L	4.48
Caudal middle frontal R	4.71	Bankssts l	4.12	Postcentral L	4.58	Supramarginal L	4.38
Rostral anterior cingulate R	4.69	Insula l	4.11	Precentral L	4.18	Postcentral L	4.3
Caudal middle frontal L	4.56	Frontal pole l	4.05	Bankssts L	3.92	Precentral L	3.81
Rostral anterior cingulate L	4.49	Medial orbitofrontal l	4.03	Pars opercularis L	3.82	Pars opercularis L	3.77
Paracentral L	4.39	Posterior cingulate r	3.96	Insula L	3.8	Superior temporal L	3.67
Frontal pole L	4.37	Paracentral r	3.88	Rostral middle frontal L	3.74	Posterior cingulate L	3.67

Abbreviations: BCI, brain–computer interfaces; ROI, region of interest.

### 3.2 Pattern classification analysis

The leave‐one‐subject‐out cross‐validation SVM pattern classification analysis (of beta‐decrement maps) provided BA rates of 74 and 68% for discriminating between the two motor‐imagery tasks (HANDS‐vs.‐FEET) and the two cognitive tasks (WORD‐vs.‐SUB) tasks, respectively. Among all possible binary classification contrasts, the highest (and most significant) accuracy rates were achieved for the HANDS‐versus‐WORD and HANDS‐versus‐SUB classifications with BAs of 85 and 80%, respectively. The poorest (and nonsignificant) classification accuracy was achieved for the FEET‐versus‐SUB contrast, with a BA of 55%. A GPC provided a BA of 60.36% for the four‐way classification problem of HANDS‐FEET‐WORD‐SUB. For ease of comparison, BAs are summarized in Table 2.

**TABLE 2:** BCI task classification accuracy using SVM and GPC classifiers. Summary classification accuracy of seven different contrasts of BCI imagery tasks. Classification analyses were conducted using SVM and GPC classifiers and using linear kernels as input features. To ensure statistical independence, an LOO cross‐validation procedure with *p*‐values generated using permutation testing with 1000 iterations was used. Class accuracy and BA (average sensitivity, and specificity of class accuracy) are reported

Contrast	Total BA (%, *p*‐value)	Class accuracy (%)	Method
HAND‐versus‐FEET	74 (.04)	[72, 76]	SVM‐LOO, Perm
WORD‐versus‐SUB	68 (.05)	[72, 65]	SVM‐LOO, Perm
HAND‐versus‐WORD	85 (.007)	[85, 85]	SVM‐LOO, Perm
HAND‐versus‐SUB	80 (.02)	[76, 78]	SVM‐LOO, Perm
FEET‐versus‐SUB	55 (.2)	[60, 50]	SVM‐LOO, Perm
FEET‐versus‐WORD	75 (.04)	[77, 72]	SVM‐LOO, Perm
HAND, FEET, WORD, SUB	60 (n/a)	[61, 64, 60, 54]	GPC‐LOO

Abbreviations: BCI, brain–computer interfaces; GPC, Gaussian‐process classifier; LOO, leave‐one‐participant‐out; SVM, support vector machine.

**FIGURE 6:**
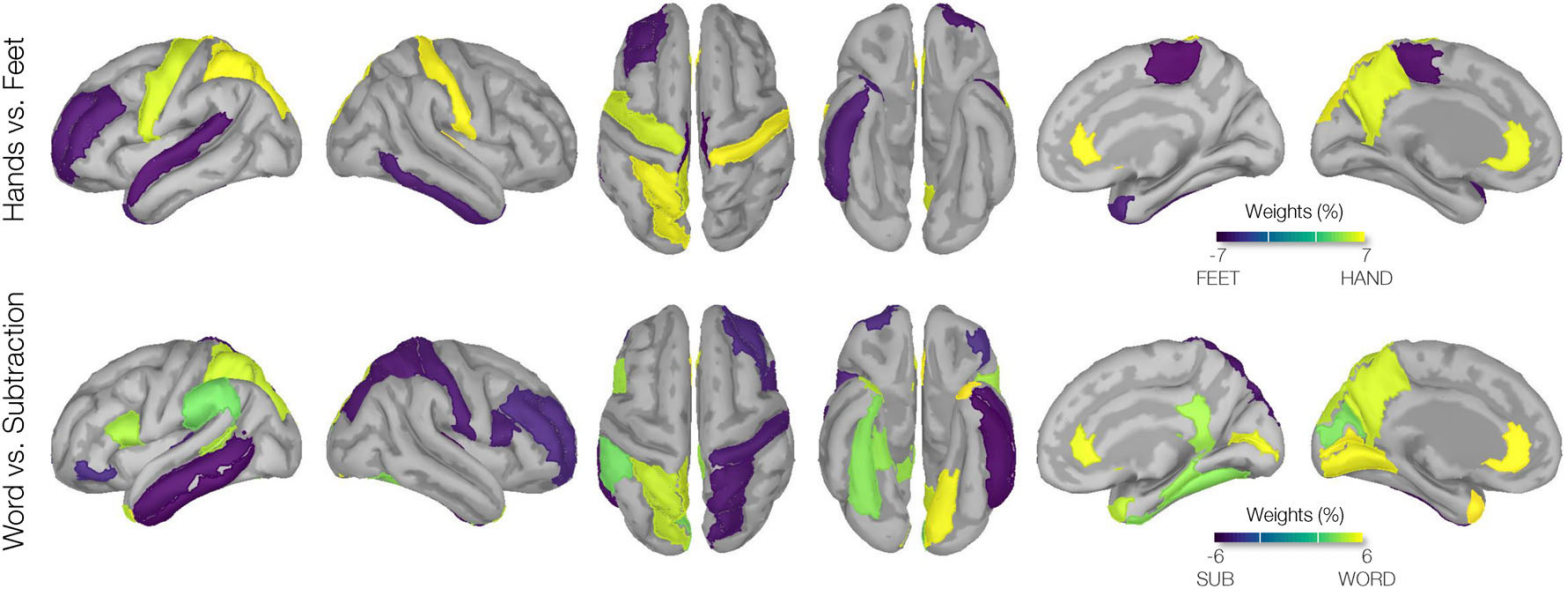
Weights (per region, Dk atlas) modeled by the support vector machine (SVM) classification of brain–computer interfaces (BCI) tasks. SVM classification weight maps of HANDS‐versus‐FEET and WORD‐versus‐SUB tasks are shown. Yellow and blue areas indicate higher SVM weights (i.e., greater beta‐power source values) toward each side of the contrast. Maps are consistent with regions reported in Table. 3. Rendered weights are displayed on a surface template (MNI‐152) with dark representing sulci and gray representing gyri. Suprathresholded values with values greater than half maximum on each side of contrast are shown. Suprathresholded regions with weights greater than an absolute value of 4 are presented.

**FIGURE 7:**
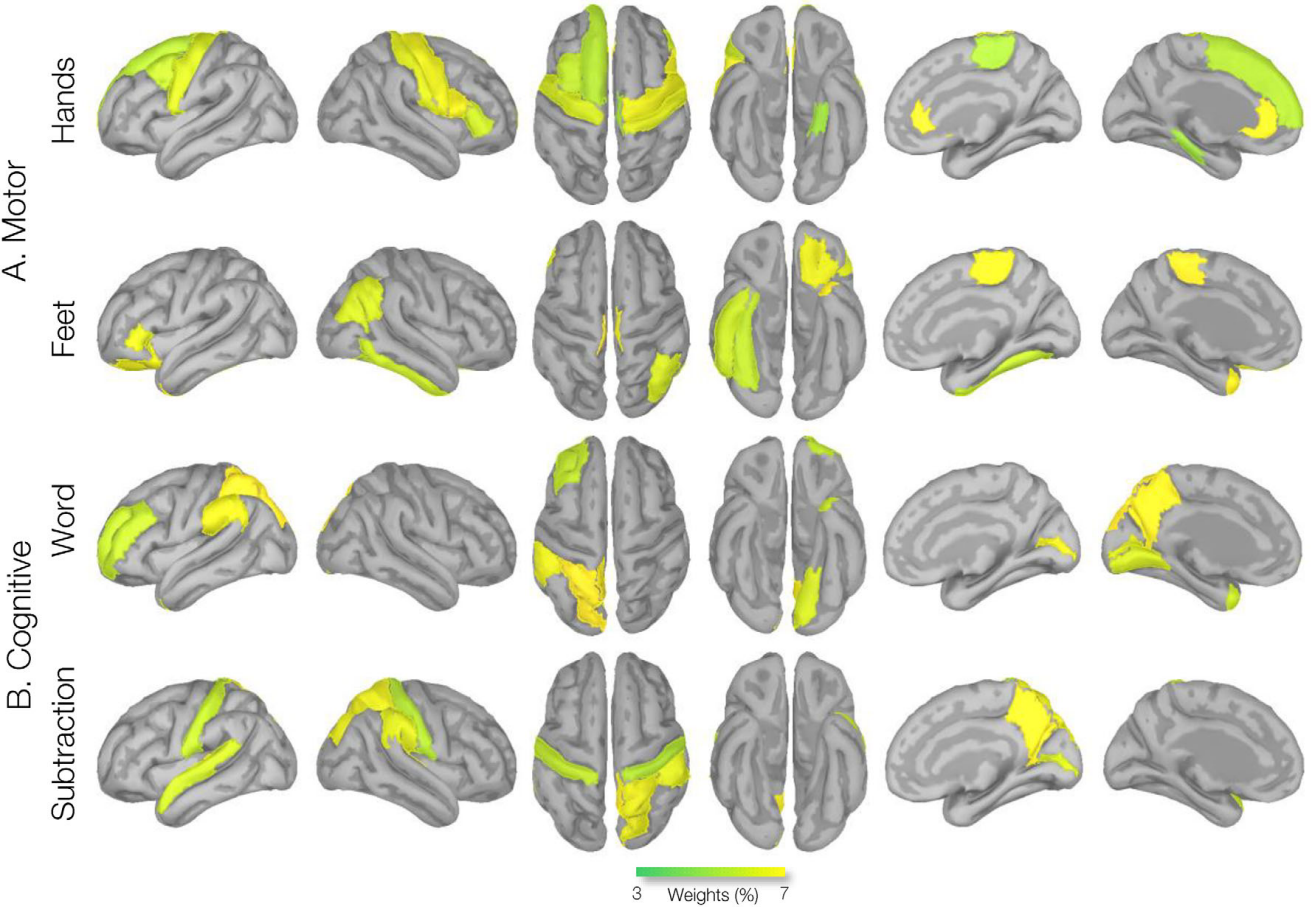
Weight (per region, Dk atlas) maps modeled by the Gaussian‐process classifier (GPC) classification of brain–computer interfaces (BCI) tasks. GPC classification weight maps of HANDS‐FEET‐WORD‐Sub tasks are shown. Maps are consistent with regions reported in Table. 4. Rendered weights are displayed on a surface template (MNI‐152) with dark representing sulci and gray representing gyri. Suprathresholded values with values greater than half maximum are shown. Regions with weight values greater than an arbitrary 3% classification weights are presented.

The SVM weight maps generated for the HANDS‐versus‐FEET classification problem showed greater contributions from paracentral regions bilaterally (SMA) for the FEET condition, and greater bilateral contributions from the central regions (right postcentral and left precentral) and rostral anterior cingulate for the HANDS task. Also, the whole‐cortex SVM weight maps generated for the WORD‐versus‐SUB classification problem revealed contributions from the left frontotemporal, and cingulate cortices (temporal lobe, anterior cingulate, supramarginal cortex, and IFG pars opercularis), and right temporal and parietal cortical regions (transverse temporal, inferior temporal, middle temporal, and superior parietal) for the SUB task conditions. Weight maps are shown in Figure 6 and ROI contributions in weight percentage are summarized in Table 3. Consistent with SVM findings, the GPC weight maps showed high contributions by bilateral (pre‐and post‐) central gyri and the anterior cingulate gyri for the HANDS task, bilateral paracentral gyri for the FEET task, left temporal, parietal, and inferior frontal regions for the WORD task, and bilateral temporal and right parietal supramarginal region for the SUB task. Weight maps are shown in Figure 7 and ROIs are reported in Table 4.

**TABLE 3:** ROI involved in SVM classification weight maps of BCI tasks. Regions contributed (in %) to the SVM classification of HANDS‐versus‐FEET and WORD‐versus‐SUB imagery tasks are reported. ROIs with percentage values greater than half‐maximum (arbitrary 50% threshold) of each side of contrast are reported

HANDS‐versus‐FEET, ROI	Weight (%)	Word‐versus‐subtraction, ROI	Weight (%)
Transverse temporal R	6.38	Temporal pole L	5.7
Rostral anterior cingulate R	5.01	Rostral anterior cingulate L	5.69
Postcentral R	4.91	Pericalcarine L	5.35
Superior parietal L	4.48	Lingual L	5.13
Rostral anterior cingulate L	4.37	Pericalcarine R	4.83
Precentral L	4.26	Rostal anteior cingulate L	4.71
Posterior cingulate L	4.19	Supramarginal L	4.31
Postcentral L	4.15	Pars opercularis L	4.22
Paracentral R	−5.93	Transverse temporal R	−5.1
Paracentral L	−5.4	Inferior temporal L	−5.0
Superior temporal L	−4.62	Middle temporal L	−4.62
Rostral middle frontal L	−4.6	Superior parietal R	−4.4
Inferior temporal R	−4.54	Postcentral R	−4.24
Temporal pole R	−4.45	Pars opercularis R	−4.01

Abbreviations: BCI, brain–computer interfaces; GPC, Gaussian‐process classifier; ROIs, region of interest; SVM, support vector machine.

**TABLE 4:** ROI involved in the GPC classification weight maps of BCI imagery tasks. Regions contributed (in %) to the GPC classification of HANDS‐versus‐FEET‐versus‐WORD‐versus‐Sub imagery tasks are reported. ROIs with percentage values greater than half‐maximum (arbitrary) are reported

HANDS, ROI	Weight (%)	FEET, ROI	Weight (%)	WORD, ROI	Weight (%)	SUB, ROI	Weight (%)
Rostral anterior cingulate R	5.42	Paracentral L	5.43	Superior parietal L	5.92	Transverse temporal L	6.34
Rostral anterior cingulate L	5.29	Temporal pole L	5.04	Precuneus L	5.62	Precuneus R	5.44
Pars opercularis R	5.13	Paracentral R	4.61	Pericalcarine R	5.26	Transverse temporal R	4.97
Precentral R	4.69	Lateral orbitofrontal L	4.41	Supramarginal L	5.01	Superior parietal R	4.86
Postcentral R	4.59	Pars triangularis L	4.01	Pericalcarine L	4.91	Supramarginal R	4.69
Precentral L	4.58	Inferior parietal R	3.84	Lingual L	4.74	Pericalcarine R	4.15
Pars triangularis R	4.43	Inferior temporal R	3.77	Temporal pole L	4.23	Superior temporal L	4.14
Caudal middle frontal L	4.35	Fusiform R	3.76	Rostral middle frontal L	4.21	Postcentral L	4.06
Paracentral R	4.3			Rostral anterior cingulate R	4.11	Postcentral R	4.0
Parahippocampal L	4.27			Paracentral R	3.99	Isthmus cingulate R	3.97

Abbreviations: BCI, brain–computer interfaces; GPC, Gaussian‐process classifier; ROIs, region of interest; SVM, support vector machine.

### 3.3 Correlation analysis

**FIGURE 8:**
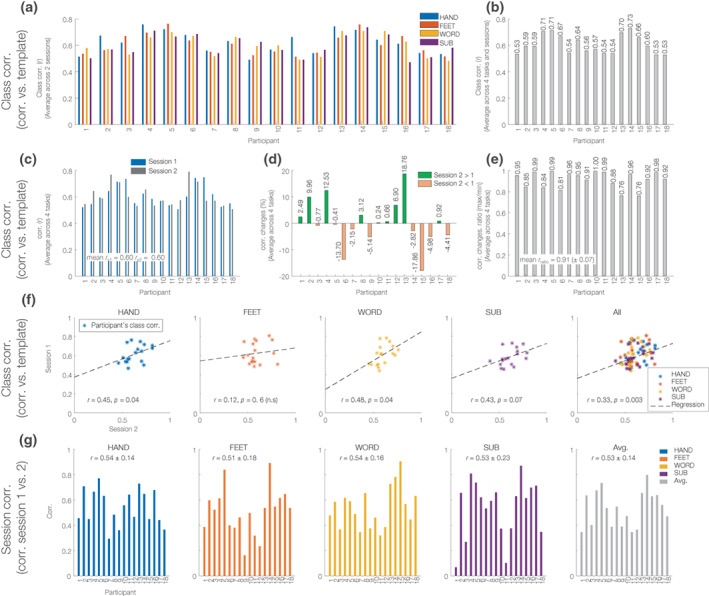
Correlation analysis of beta‐decrements in brain–computer interfaces (BCI) tasks. A Spearman correlation of beta‐decrements of (a) four different imagery tasks (b) completed by 18 participants during (c) two sessions of a BCI experiment. (d) Class correlation changes between sessions 1 and 2. The green and orange bars indicate increased and decreased correlation values for session 2 compared with session 1 of the task. (e) Beta‐decrement correlation ratio. The correlation ratio was computed by dividing the maximum over the minimum of individual class correlations between two sessions (f) scatterplots of class correlations of four BCI tasks. A linear Spearman correlation between sessions 1 and 2 is also reported, and a polynomial curve fitting line is overlaid. (g) Session‐correlations, the correlation between sessions 1 and 2 of four BCI task conditions. Average and standard deviation session correlations are also shown and reported.

Correlations of individual beta‐decrement maps and templates defined by the class mean (averaged across tasks and sessions) ranged between 0.53 and 0.73 (Figure 8b), with subjects 14, 4, 5, and 13 showing the highest correlations of *r* = .73, .71, .71 and .70, respectively. Across the two task sessions, participant 13 showed the most improved correlations (18%) from sessions 1 to 2, but this improvement was not always the case (not seen in, e.g., participants 15 and 6 with 17 and 13% decline in correlations, respectively). The correlations between a subject's beta‐decrement map for a task and the class‐mean template averaged across all four tasks were *r* = .60, in both sessions 1 and 2. A high ratio of correlations to the class‐mean template, averaged over tasks, between sessions 1 and 2 (mean = 0.91, SD = 0.07) suggests consistency of cortical engagement across sessions. The correlation to class‐mean showed significant positive correlations between sessions for WORD and HAND tasks (*r* = .48, *p* = .04 and .45, *p* = .04), respectively, whereas the SUB and FEET tasks showed nonsignificant correlations of *r* = .43 and .12, respectively. This suggests that the beta‐decrement maps for SUB and FEET tasks may be more unstable. Direct correlations of the beta‐decrement maps across sessions for individual subjects (Figure 8g) appear to support this inference: they show the highest values for WORD and HAND task conditions (*r* = .54 for both), and the lowest for the SUB and FEET task conditions (*r* = .53 and .51, respectively). Figure 8 summarizes these findings.

## 4 DISCUSSION

This study set out to characterize and localize the neural correlates of motor imagery and mentally executed cognitive tasks in a group of healthy adult participants who completed an experimental paradigm that was designed for BCI research. We localized task‐related cortical engagement from beta power decrements (Youssofzadeh et al., 2020) during motor imagery of the hands and feet, mental arithmetic (subtraction), and silent word generation. Our source‐space analyses at the group level revealed bilateral beta‐desynchrony effects during motor imagery, notably in the motor region (pre‐, post‐, and para‐central), the anterior portions of the cingulate gyri, middle‐frontal gyri, and parts of the temporal neocortex. Group‐level beta‐decrements in the source space during the two cognitive tasks revealed left‐lateralized effects in the parietal, temporal, and (inferior) prefrontal cortical regions.

Specifically, cortical engagement during motor imagery of the hands revealed bilateral beta power decrements in the SMA and the precentral/primary motor cortex (BA 4), as well as anterior cingulate gyri (caudal and rostral) and prefrontal (caudal and medial orbitofrontal and frontal pole) regions (Figures 4a and 5a and Table 1). The SMA, precentral, and postcentral gyri have both motor and sensory representations related to both upper and lower limbs (Jenkinson & Brown, 2011; Szameitat et al., 2007). The SMA is believed to support aspects of movement planning, including action preparation and the organization of movement sequences, and appears to have a role in the perception of stimuli that are potential targets of motor acts, and has been found to engage during cued movements (Bonini et al., 2014; Hardwick et al., 2013; Lima et al., 2016; Matsuzaka et al., 1992; Nachev et al., 2008). The prefrontal cortex areas integrate information from the body and the environment and participate in higher‐order gait control (Maidan et al., 2016; Van der Meulen et al., 2014). In particular, the medial frontopolar prefrontal cortex (BA 10) is involved in the integration of spatial and motor components of working memory during imagery and haptic exploration of spatial layouts, guiding motor preparatory processes (Kaas et al., 2007). We also found strong activation in the (caudal and rostral) anterior cingulate cortex (ACC) regions. The ACC (BA 32) lies in a unique position in the brain with connections to both the “emotional” limbic system and the “cognitive” prefrontal cortex (Bush et al., 2000). The ACC regions were shown to be engaged in a BCI motor imagery task requiring focused visual attention (Luu & Posner, 2003; Pfurtscheller et al., 2006). The ACC is also believed to play an important role in attentional control through bidirectional interactions with primary sensory areas (Crottaz‐Herbette & Menon, 2006; Kim et al., 2016).

Group‐level source analysis during motor imagery of the feet showed prominent beta power decrements in the paracentral/SMA and prefrontal (lateral orbitofrontal) regions; it also showed bilateral engagement of temporal regions (insula and superior temporal sulcus). The paracentral, precentral, and postcentral gyri also feature motor and sensory functions related to the lower limbs (Jenkinson & Brown, 2011). The paracentral lobules have the motor and sensory representations of the contralateral lower extremities, with sensory representations in their posterior portion (in the parietal lobe). Activation in paracentral/SMA has been found in studies of actual walking, using single‐photon emission computed tomography and near‐infrared spectroscopy (Fukuyama et al., 1997; Miyai et al., 2001). The superior temporal sulcus is a voice‐selective area in the human auditory cortex and a source of sensory encoding associated with motor output through the superior parietal–temporal area (Belin et al., 2018). The anterior insula has been shown to be involved in mental navigation along memorized routes; it also supports the feeling of agency, awareness of body parts, and self‐awareness (Craig, 2009; Ghaem et al., 1997; Van der Meulen et al., 2014). The group‐level cortical engagements that we find during motor imagery of the feet are generally consistent with the results of the previous fMRI studies on gait imagery and limb movement imagery, supporting engagements in the primary and supplementary motor cortices as well as bilateral parietal and frontal areas (Bakker et al., 2007; Bakker et al., 2008; Cojan et al., 2009; la Fougère et al., 2010; Van der Meulen et al., 2014; Wang et al., 2008).

Findings for the word generation and subtraction tasks showed strong left‐hemispheric beta‐decrement effects in the temporal (superior temporal gyrus), parietal (supramarginal gyrus), and (inferior) prefrontal gyri (Figures 1b and 4b), regions known to be involved in cognitive (language) and comprehension (arithmetic) processing (Arsalidou et al., 2018; Arsalidou & Taylor, 2011; Binder & Desai, 2011; Koyama et al., 2017; Patterson et al., 2007). The supramarginal gyrus is an anatomical subdivision of the inferior parietal lobule, a heterogeneous brain region involved in the interpretation of both sensory and language information (Cabeza et al., 2008; Corbetta & Shulman, 2002; Dehaene et al., 2004). The left and right SMG regions are engaged in phonological (word) processing, while the left SMG is more engaged in the semantic processing of lexical items (Hartwigsen et al., 2010; Oberhuber et al., 2016). Moreover, both cognitive tasks revealed desynchrony effects in the IFG and postcentral (premotor) areas. The inferior parietal lobule is anatomically connected to ventral premotor areas, and the caudal inferior parietal gyrus is connected to the IFG regions (Caspers et al., 2013; Petrides & Pandya, 2009). Unlike the Word (linguistic) task, the Subtraction task led to greater beta‐power decrease effects in the left superior parietal lobule (SPL), whereas the Word condition task showed greater beta‐power decrease effects in the left temporal regions, the inferior temporal gyrus (ITG), and the middle temporal gyrus (MTG). In general, mathematical calculations strongly engage the working memory, and the SPL is critically important for manipulating and rearranging information in the working memory processes (Bemis & Pylkkänen, 2013; Koenigs et al., 2009; Roitman et al., 2012). The ITG and its neighboring region, MTG, provide access to lexical‐semantic representations during concept retrieval processes (Binder et al., 2009; Hickok & Poeppel, 2007; Schuhmann et al., 2012).

Our results from the pattern classification analyses demonstrate that beta‐decrement mapping is a suitable approach for identifying cortical engagement related to BCI tasks from MEG recordings. Specifically, the SVM classification analysis of the BCI imagery task completion provided a substantially high classification accuracy for two‐way classification problems of HANDS‐versus‐WORD (85%), HANDS‐versus‐SUB (80%), HANDS‐versus‐FEET (74%), and WORD‐generation‐versus‐SUBtraction (68%). Also, the GPC four‐way classification accuracy of 60% without any further dimension‐reduction is reasonable given the complexity of combined task imagery responses. For our machine‐learning pattern classification analyses, we utilized simple linear kernel values to train and classify four complex BCI problems. Generally, kernel methods are extremely useful for fast and efficient analyses and avoid intensive computations. In addition to the computational advantages, kernels enable the solution of ill‐conditioned problems and therefore avoid overfitting (Shawe‐Taylor & Cristianini, 2004). The application of kernel methods to neuroimaging problems has been growing (Chu et al., 2011; Schrouff et al., 2015; Youssofzadeh et al., 2017) and may be a potential candidate for real‐time BCI applications.

The correlation analysis of beta‐decrement effects reveals the consistency of responses between the two sessions in individual subjects, which may be related in part to the level of engagement with task imagery. For instance, higher class correlations were obtained for three participants, 14, 4, and 5, which may suggest that these participants likely had better overall performance than others. This should ideally have been supported by behavioral data, and the lack of such measurements in the experimental paradigms that were employed is a key limitation of our study. This should be addressed in the design of future BCI paradigms (Klepp et al., 2015). Our correlation analysis indicated greater consistency of correlation to the group mean for the WORD and HAND imagery conditions (*r* = .48 and .45, respectively) compared to the SUB and FEET tasks (*r* = .43 and .12, respectively), as shown in Figure 8f. These findings are consistent with the lower (55%) binary classification accuracy of FEET‐versus‐SUB and a high (85%) classification accuracy of HAND‐versus‐WORD contrasts, as reported in Table 2, as well as the lower average session‐to‐session correlation of cortical‐engagement maps for these two conditions (Figure 8g) suggesting that mapping of mental subtraction and motor imagery involving the feet may be significantly less reliable than word generation and hand movement imagery tasks using beta‐decrement effects.

Recent years have seen the increasing use of neuroimaging methods in the context of BCI research. Several studies have investigated the feasibility of MEG in real‐time neurofeedback experiments (Boe et al., 2014; Buch et al., 2008; Florin et al., 2014; Foldes et al., 2015; Fukuma et al., 2015; Fukuma et al., 2016; Gerven et al., 2009; Mellinger et al., 2007; Okazaki et al., 2015). These studies have used both sensor and source‐level MEG signals to provide feedback aimed at modulating specific brain activities. Indeed, the high temporal and spatial resolution of MEG makes it inherently suitable for real‐time applications. Most BCI studies rely on the early P300 or N400 ERP components to examine the sources of task‐related cortical engagement. However, brain source activities of late components are usually not phased‐locked to an event and, therefore, cannot be extracted by linear methods such as averaging; they, however, may be detected by power changes in the frequency domain (David et al., 2006; Pfurtscheller & Andrew, 1999). ERP source estimates are limited to slow responses due to the effects of signal averaging in the time domain. By contrast, induced power changes in the beta or gamma bands can serve as signatures of task‐related cortical engagement (Crone et al., 2006; Eulitz et al., 1996; Singh et al., 2002). Beta‐band power decrements have been used for classification purposes and clinical rehabilitation, for example, characterization of motor or cognitive impairments as a therapeutic marker for patients recovering from a cerebral stroke (Buch et al., 2008) or helping robot‐assisted gait training of patients with Parkinson's disease or motor disability (Gwin et al., 2011; Knaepen et al., 2015; Severens et al., 2012).

The beta‐decrement approach can be applied to not only MEG and low‐cost EEG systems but also the relatively new technology of optically pumped magnetometers that offer good signal‐to‐noise ratio, spatial resolution, and portability for measuring BCI task activities (Boto et al., 2018; Knappe et al., 2012). The ultra‐high‐density EEG system is another relatively new technology where beta decrements have shown excellent decoding accuracy for a finger movement BCI task (Lee et al., 2022). Comparing the two studies, the focus of our work was mainly to localize neural activity during mixed (motor and mental) imagery task responses and decode source activations, whereas the work by Lee et al. (2022) focused on motor task activations at the channel level. MEG is less affected by sources of interference from outside the head, and due to its better spatial resolution, sources of brain activity can be localized more accurately in the brain. This is important in BCI applications, where it is critical to determine the location of specific brain signals associated with motor or mental tasks. However, as suggested by the authors in the discussion section of Lee et al. (2022), source reconstruction provides better reliability for decoding and analyzing high‐density EEG data. Ultra‐high‐density EEG technology has great potential for BCI applications, particularly in individuals with neurological impairments, and future studies may benefit from combining EEG and MEG data to improve the accuracy and localization of BCI systems.

The current study aimed to localize the neural activity during mixed imagery task responses, for which the beta frequency power decrements may be a suitable marker of cortical engagement. Interestingly, other previous BCI studies have supported the use of upper‐alpha/beta‐desynchronization effects for the characterization of the MI responses, also suggesting such oscillations reflect the search and retrieval processes in semantic long‐term memory processes (Klimesch, 1999; Pichiorri et al., 2015). The use of other frequencies, such as theta‐band (4–8 Hz) peaks, which reflect the encoding of new information (episodic memory), in conjunction with beta‐band source modeling, has the potential to improve the understanding of the underlying neural dynamics of BCI tasks and may lead to improved subjects' decoding accuracy in real‐time BCI tasks. As a cautionary note, beta‐decrements may be affected by carryover effects between trials when using pre‐stimulus data as a baseline contrast. While such influences can be minimized by including a high number of trial responses as we did in this study, an additional step to resolve this could be adding a control condition to the BCI experiment design or constructing the baseline from pre‐stimulus data of all data conditions. We hope to incorporate and test the suggested changes into our future research investigations.

Our classification analysis was based on whole‐brain beta‐decrement activations. While this allows for investigating widespread activators, it may be less efficient for decoding purposes. In future work, exploring beta‐decrements at optimal ROIs for a particular task may effectively improve the accuracy of the decoding analysis. Another limitation is that our approach does not offer individual classification. The kernel‐based classification analysis incorporates the group cross‐similarity of task beta‐decrements as input features. To address this, we conducted a univariate correlation analysis of beta‐decrements against the average template. However, more suitable multivariate pattern recognition approaches utilizing optimal features may be used for individual classification purposes (Roy et al., 2020). Finally, our analyses were primarily designed to classify single‐trial beta‐decrement maps (i.e., a summary of 4‐s task trials). Some modifications are required for real‐time implementations. For source modeling, a pre‐estimated spatial filter from individuals' training sessions can be used, and for computational efficiency, classification can be conducted at optimal ROIs (informed by the group analysis). Utilizing cutting‐edge classification methods like deep learning may improve the decoding accuracy of real‐time BCI applications (Lotte et al., 2018; Lotze & Cohen, 2006).

One limitation of our BCI task was that we did not control for the possibility of actual movement during movement imagery. A control task would be ideal to ensure that the responses being studied are indeed related to movement imagery alone. The persistence of visual cues during our BCI task may also be an undesirable feature since it could lead to habituation to the cues and potentially distract from the task (Lacourse et al., 2004). While such effects are likely to affect the alpha‐ and lower beta‐band responses more than the higher beta‐band power activities (17–25 Hz), which were the focus of this study, paradigms that present visual cues only when needed rather than continuously throughout the task are more suitable to maintain the participant's attention and focus on the task. Another limitation of the current dataset is the lack of behavioral and performance measures. The ability to imagine the task is a complex cognitive process that involves creating an internal representation of a motor act in the mind without actually performing the movement. Several components are considered essential in assessing this ability, including visual imagery (the ability to form a vivid and clear mental image of the movement), kinesthetic imagery (the ability to feel and experience the sensory‐motor aspects of the movement, such as the movement's force, speed, and direction), temporal imagery (the ability to experience the movement in time, including the duration and rhythm of the movement), and mental imagination (the ability to simulate the movement in real‐time, as if the movement were actually being performed). These components are often evaluated through self‐reported questionnaires or rating scales, behavioral performance measures, and neurophysiological measures of M/EEG. Extensive studies have investigated the neurophysiological markers of motor imagery as well as the neural mechanisms underlying BCI learning and performance (Ahn, Ahn, et al., 2013; Ahn, Cho, et al., 2013; Ahn & Jun, 2015; Bamdadian et al., 2014; Blankertz et al., 2010; Corsi et al., 2020; Grosse‐Wentrup et al., 2011). Specifically, Corsi et al. (2020) compared the motor imagery and rest conditions in four BCI sessions and reported a progressive decrease in α and β‐band functional connectivity of M/EEG activations in regions of the middle‐anterior cingulate gyrus and orbital IFG known to be associated with decision‐making and memory consolidation processes. We hope to address such mechanisms in a future dataset that includes behavioral measurements and measurements from multiple training sessions.

In summary, our results demonstrate the feasibility of using oscillatory dynamics of MEG signals, particularly beta‐band power decrements, for localizing cortical engagement during a set of tasks designed for BCI research that did not involve dynamic sensory stimuli or overt behavioral responses. These motor imagery and mentally executed cognitive tasks (Rathee et al., 2021) engaged task‐specific networks of brain regions that were largely consistent with prior neuroimaging studies of similar tasks in healthy adults and known neuroanatomy. Our results lend further support to the idea that task‐related beta‐band power decrements are closely associated with neural engagement in the cerebral cortex (Engel & Fries, 2010; Pfurtscheller & Lopes da Silva, 1999) and may be suitable for MEG BCI applications. BCI techniques have been successfully used in cognitive and motor training, leading to improvements in the performance of athletes, musicians, and highly skilled manual technicians such as surgeons (Meister et al., 2004; Rogers, 2006; Toth et al., 2020), as well as those with stroke, cerebral palsy, severe physical disability, or motor impairment (Aflalo et al., 2015; Baniqued et al., 2021; Cincotti et al., 2012; Machado et al., 2010; Pfurtscheller et al., 2009). Specifically, Aflalo et al. (2015) used a microelectrode BCI system and demonstrated that motor imagery is supported by a distributed network of brain regions, including the posterior parietal cortex, and that this network is functional even in individuals with severe physical disabilities. Their results suggest that it may be possible to decode motor imagery from the brain and use it to control external devices, such as prosthetics, in individuals who have lost the ability to move their limbs. In line with these studies, our results suggest that beta‐band power decrements may have potential clinical applications for communication and rehabilitation by localizing task‐related cortical engagement.

## AUTHOR CONTRIBUTIONS

Vahab Youssofzadeh and Sujit Roy conceived the idea and developed the methods. Girijesh Prasad oversaw data acquisition. Sujit Roy organized the data. Vahab Youssofzadeh performed the visualization and analysis. Vahab Youssofzadeh, Sujit Roy, Lauri Parkkonen, and Girijesh Prasad validated the analysis. Vahab Youssofzadeh, Sujit Roy, and Manoj Raghavan drafted the manuscript. All authors participated in critically reviewing and revising the manuscript.

## CONFLICT OF INTEREST

The authors declare no conflict of interest.

## Data Availability

MEG data were analyzed using a combination of FieldTrip v20190419 (fieldtriptoolbox.org) and Brainstorm v060320 (neuroimage.usc.edu/brainstorm) toolboxes in MATLAB 2019a (The Mathworks, Inc.). Custom Matlab scripts, including a Brainstorm implementation of nonoverlapping temporal windows for DICS beamformer source analysis, are available at github.com/vyoussofzadeh/BCI-beta-desynchrony_2021. All machine learning modeling steps were performed using PRoNTo v2 (mlnl.cs.ucl.ac.uk/pronto).
